# USE OF PRIMARY HEALTHCARE SERVICES BEFORE AND AFTER SPECIALIZED REHABILITATION AND ITS RELATION TO CHANGES IN HEALTH AND FUNCTIONING: A LONGITUDINAL COHORT STUDY

**DOI:** 10.2340/jrm.v56.39912

**Published:** 2024-08-20

**Authors:** Anne Mette BERGET, Vegard Pihl MOEN, Merethe HUSTOFT, Jörg ASSMUS, Liv Inger STRAND, Jan Sture SKOUEN, Øystein HETLEVIK

**Affiliations:** 1 Centre of Habilitation and Rehabilitation in Western Norway, Haukeland University Hospital, Bergen, Norway; 2 Department of Global Public Health and Primary Care, University of Bergen, Bergen, Norway; 3 Department of Health and Functioning, Western Norway University of Applied Sciences, Bergen, Norway; 4 Centre for Clinical Research, Haukeland University Hospital, Bergen, Norway; 5 Department of Physical Medicine and Rehabilitation, Haukeland University Hospital, Bergen, Norway

**Keywords:** rehabilitation, functioning, health, primary healthcare, general practitioner, physiotherapy, secondary healthcare, specialist healthcare

## Abstract

**Objective:**

To examine patients’ use of primary healthcare (PHC) before and after specialized rehabilitation and its relation with self-reported health and functioning.

**Design:**

Longitudinal cohort study.

**Participants:**

451 rehabilitation patients.

**Methods:**

Register data were used to measure the frequency of visits to the general practitioner (GP) and physiotherapist (PT) in PHC 3 years before and after rehabilitation. Patients reported health (EQ-VAS) and functioning (SF-36) before rehabilitation and at 1 and 3 years after. Data are described for the total study cohort and subgroups with musculoskeletal disease (MSD) and cardiovascular disease (CVD).

**Results:**

There was an increase in GP and PT visits preceding rehabilitation and a gradual decrease thereafter. An exception was GP visits among patients with CVD, with few diagnosis-specific visits before but an increase after. Lower levels of health and functioning tended to be related to more frequent GP and PT visits. An indication of clinically important improvement was found among those with frequent GP visits in the MSD subgroup, and among those with 1–2 GP visits in the CVD subgroup.

**Conclusions:**

The diverse relationship between health and functioning, and the use of PHC services at follow-up, may imply that additional factors besides healthcare use explain long-term improvement following rehabilitation.

## LAY ABSTRACT

In this study, we included 451 rehabilitation patients and examined their use of primary healthcare 3 years before and after specialized rehabilitation and its relation with self-reported health and functioning. We found an increase in visits to the general practitioner (GP) and physiotherapist (PT) leading up to rehabilitation and a gradual decrease thereafter. An exception was GP visits among patients with cardiovascular diagnoses. They had few visits specific to the diagnosis before rehabilitation followed by an increase after. There seemed to be a trend that patients with lower levels of self-reported health and functioning had a higher frequency of primary healthcare use after rehabilitation. The group with frequent GP visits in the musculoskeletal subgroup and those with 1–2 GP visits in the cardiovascular subgroup had a tendency for greater clinically important improvement. Results may imply that additional factors besides healthcare use explain long-term improvement following rehabilitation.

Regardless of diagnoses, rehabilitation aims to optimize functioning (1). Functioning is seen as an indicator of health, including both biological health and the lived experience of health in the context of the individual’s environment (2, 3). Rehabilitation is provided within primary (PHC) as well as secondary healthcare (specialist healthcare). Individuals referred to specialized rehabilitation are generally in need of more specialized or intensive rehabilitation than provided in PHC (4).

A strong PHC is considered essential to achieve universal health coverage and better patient outcomes (5, 6). PHC includes health promotion, disease prevention, and treatment provided by general practitioners (GPs), nurses, occupational therapists, and physiotherapists (PTs), among others. As the first-line healthcare, it is considered essential to ensure physical and mental functioning across diagnostic groups and health problems (7). In several countries, such as Norway, GPs are gatekeepers to specialist healthcare (4).

The use of healthcare services may vary between disease groups. Patients with musculoskeletal diagnoses (MSD) are commonly high users of healthcare services at both levels (8, 9). A Danish register study among 2,929 individuals with chronic musculoskeletal pain found that 40% coped without healthcare during an observational period of 10 years, whereas 8% had consistent high use of healthcare across levels. The use of rehabilitation was almost exclusively found within the subgroup of high healthcare users (10). There is evidence that rehabilitation improves functioning across diagnoses but that the effect tends to diminish over time (11, 12). It is suggested that lack of follow-up may be a contributing factor for this, emphasizing the vulnerability of the transition of care between healthcare levels (13). Continuation of care across levels is recognized as important to ensure prolonged effect (14). A recent Norwegian study among patients with rheumatic and MSD found that 98% reported a need for follow-up in PHC after specialized rehabilitation. However, only 56% reported that follow-up was planned at discharge (14). The potential value of follow-up has been shown in another Norwegian study (15). This study found that patients with severe disability after stroke maintained or improved their functioning with long-term follow-up by the PT in PHC after specialized rehabilitation (15). Follow-up from GPs and PTs is reported as the most important healthcare service by patients with rheumatic and MSD (15). However, the extent of healthcare services received by the individual may vary, depending on the diagnosis and course of illness, and little is known regarding the use of PHC services before compared with after specialized rehabilitation.

We need more knowledge regarding treatment trajectories for patients referred to specialized rehabilitation. Accordingly, the aims of this study are (*i*) to explore the trajectory of PHC 3 years before and after specialized rehabilitation among a heterogeneous group of patients and for subgroups with MSD and cardiovascular diagnoses (CVD); (*ii*) to investigate whether the frequency of PHC services used by the patient during the first 6 months following rehabilitation is related to (a) the patient’s self-reported health and functioning, and (b) clinically important change (improvement, no change, deterioration) in self-reported health and functioning.

## METHODS

### Study design

This longitudinal cohort study is part of the Rehabilitation Cohort West study (REKOVE). REKOVE is a multicentre, longitudinal cohort study conducted among a heterogeneous group of patients accepted for specialized rehabilitation and is based on patient-reported survey data and register data.

### Participants

**Fig. 1:**
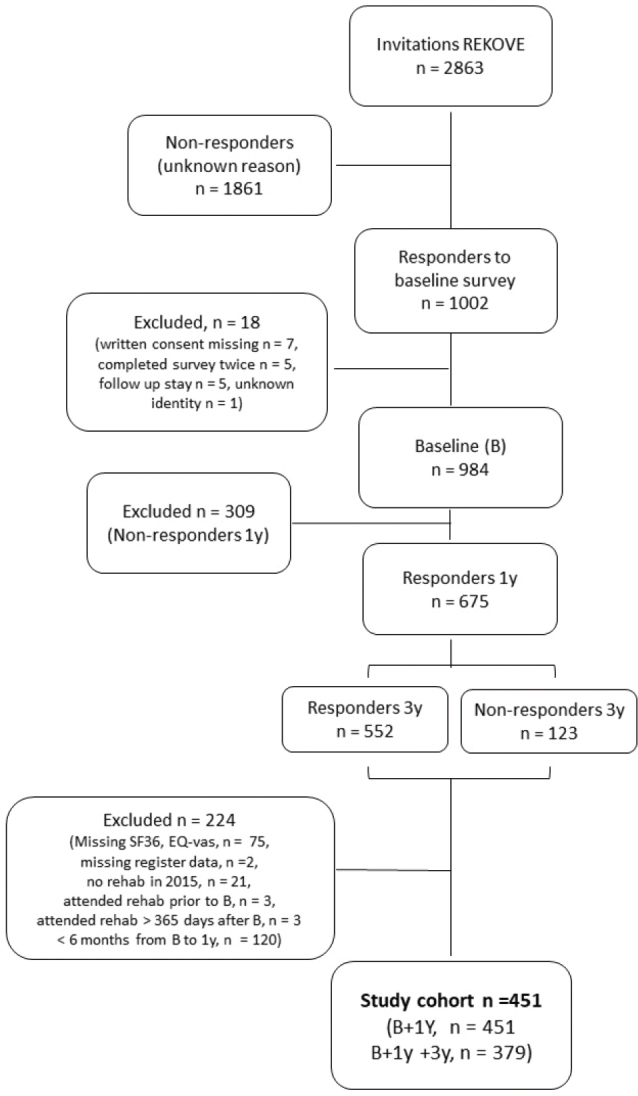
Flowchart of the inclusion process. Invited participants lived in Western Norway, were 18 years or above and accepted for specialized rehabilitation in January–June 2015. REKOVE: the Rehabilitation cohort west study; B: baseline; 1 y: 1 year; 3 y: 3 years; SF-36: the Medical Outcome Study Short Form 36; EQ-VAS: EuroQol visual analogue scale.

Patients across diagnoses, aged ≥ 18 years, living in Western Norway and accepted for specialized rehabilitation in secondary healthcare between January and June 2015 were invited to participate in the REKOVE study. In the present study, all patients who participated in the baseline survey and in follow-up surveys were included. Those who had completed neither the EuroQol visual analogue scale (EQ-VAS) nor the Medical Outcome Study Short Form 36 (SF-36) or had missing register data were excluded. Additionally, patients who had not attended rehabilitation in 2015, had attended rehabilitation before responding to the baseline survey, attended rehabilitation > 365 days after baseline or had < 6 months between baseline and the 1-year follow-up survey were also excluded (Fig. 1).

All the eligible participants received up to 4 weeks of somatic, interprofessional rehabilitation as an inpatient or outpatient in specialized rehabilitation centres between baseline in 2015 and the 1-year follow-up survey in 2016. The operating agreement between the 6 rehabilitation centres and the regional health authority states that rehabilitation interventions should be goal oriented, individually adapted, and focused on physical activity, cognitive approaches, coping strategies, and pain management. This study did not investigate the interventions provided.

### Data collection

Patient-reported data were collected preceding rehabilitation in 2015 (baseline), and follow-up data were collected in 2016 and 2018. Register data (2012–2018) were retrieved from Statistics Norway (SSB), the Norwegian Control and Payment of Health Reimbursements database (KUHR), and the Norwegian patient register (NPR). KUHR data included contacts with GPs and PTs (with municipal operating agreements). The NPR contains data on the time for the start and end of rehabilitation stays in secondary healthcare. From SSB, we retrieved sociodemographic data (age, gender, level of education). Patient-reported data from the surveys were linked to data from KUHR, NPR, and SSB using a project-specific ID generated at SSB, based on the national person ID number.

### Variables

*Physical and mental functioning* were measured in 2015, 2016, and 2018 using the SF-36, version 1. In SF-36, 8 functional domains are measured and summarized into two components: (*i*) the physical component summary (PCS) measuring general health, bodily pain, physical functioning, and role physical, and (*ii*) the mental component summary (MCS) measuring vitality, social functioning, mental health, and role emotional (16). In accordance with the SF-36 scoring manual, both PCS and MCS scores were calculated on a scale from 0 to 100, with a higher score indicating better functioning (16, 17). To obtain PCS and MCS scores, participants had to respond to at least 50% of the items (17). The SF-36 has been found to be a valid and reliable instrument (18). Reference values for the Norwegian population are available (19). Three categories (improvement, no change, deterioration) were used to describe change from baseline to 1 and 3 years, based on the minimal important change (MIC) threshold of 3 points (≥ 3 points = improvement; ≥ –3 points = deterioration) recommended for SF-36, version 2 (20, 21).

*Health* was measured using the EQ-VAS in 2015, 2016, and 2018. The EQ-VAS measures the patients’ overall health ranging from zero (“worst imaginable health state”) to 100 (“best imaginable health state”) (22). It is a validated instrument (23), and reference values for the Norwegian population are available (24). Permission to use this instrument was obtained by the EuroQol group. Three categories (improvement, no change, deterioration) were used to describe change from baseline to 1 and 3 years, based on a MIC value of 11 points (≥ 11 points = improvement; ≥ –11 points = deterioration), as was used in a recent Swedish study (25).

*The frequency of GP and PT visits* was measured using data from KUHR and NPR. All GPs and most PTs in PHC have municipal operating agreements and receive part of their payment based on fee-for-services by sending claims for each patient contact (stored in KUHR). To each claim, the GP adds a diagnosis using the International Classification of Primary Care version 2 (ICPC-2), while this is not part of the claims from the PT.

For GPs, we included consultations with the reimbursement codes 2AD, 11AD, 11AK, 2AK, and 2AE from KUHR, denoted “visits”. Additionally, the diagnosis code was used to define whether the visits were related to MSD (L chapter in ICPC-2) or CVD (K chapter in ICPC-2). The following variables were generated:

- Number of visits to the GP per 6-month period between 3 years before and after rehabilitation. All visits to the GP were calculated for the total study cohort. For the subgroups, visits related to the referral diagnosis (MSD/CVD) were calculated.
- Time to the first contact with the GP was defined as the number of days from the date participants were discharged from rehabilitation to the date of the first visit (for all and visits related to MSD and CVD).
- Number of contacts during the first 6 months after rehabilitation summarized from the date participants were discharged and categorized into 4 categories (0, 1–2, 3–4, ≥ 5 visits), calculated for all and related to the referral diagnoses for the MSD and CVD subgroups.

All consultations with the PT involving direct patient contact, including treatment in groups, were used to define PT visits; thus, delimited administration codes (“E” codes) were omitted. The number of visits to PT per 6-month period before and after rehabilitation, time to first contact, and number of contacts were defined as for GP (see above). The number of PT visits was categorized into 4 categories (0, 1–7, 8–12, ≥ 13).

### Statistics

We used descriptive methods to analyse the data, reporting percent, mean, and standard deviation (SD) in addition to graphic presentations. The data were prepared and described using Stata/SE 17 for Windows (StataCorp LLC, College Station, TX, USA), R 4.2.2 (26) and MATLAB (MathWorks Inc., Natick, MA, USA).

## RESULTS

Of the 984 patients participating in the REKOVE study, 451 met the inclusion criteria for the present study (Fig. 1). Among the included participants, 451 responded to the baseline and the 1-year follow-up survey, and 379 responded to all 3 surveys (baseline, 1 year, and 3 years). The mean (SD) age was 59 (13) years, 59% were women, 47% (*n* = 210) had MSD, and 22% (*n* = 99) had CVD (Table I).

**Table I:** Characteristics of the 451 participants aged 18 years or above, included in the study

Category	Total study cohort		Subgroups, diagnoses			

	*n*	Value	Musculoskeletal		Cardiovascular	

			*n*	Value	*n*	Value
Age at baseline in years, mean (SD)	451	58.6 (13.3)	210	57.1 (14.0)	99	61.9 (9.4)
Sex (female)		264 (58.5%)		155 (73.8%)		28 (28.3%)
Place of residence (urban)		225 (49.9%)		104 (49.5%)		56 (56.6%)
Level of education	449		208		99	
University/college		141 (31.4%)		68 (32.7%)		24 (24.2%)
Upper secondary school		231 (51.5%)		100 (48.1%)		61 (61.6%)
Primary school		77 (17.2%)		40 (19.2%)		14 (14.1%)
Referral diagnoses	451		210		99	
Musculoskeletal		210 (46.6%)		210 (100%)		
Cardiovascular		99 (22.0%)				99 (100%)
Neoplasm		23 (5.1%)				
Neurological		32 (7.1%)				
Other		87 (19.9%)				

The MSD subgroup was younger and included more women (74%) than the CVD subgroup (women = 28%). The mean PCS, MCS, and EQ-VAS scores of the total study cohort and both subgroups were below the mean scores of the Norwegian reference data (Table II).

**Table II:** Physical (PCS) and mental (MCS) functioning (SF-36) and EQ-VAS as reported by the 451 participants in 2015 (b), 2016 (1 y) and 2018 (3 y)

	SF-36						EQ-VAS		

	PCS			MCS			*n*	Mean (SD)	Min–max

	*n*	Mean (SD)	Min–max	*n*	Mean (SD)	Min–max			
Study cohort									
b	451	33.4 (9.7)	9.8–63.9	451	44.9 (11.7)	8.9–70.9	451	51.4(18.7)	0–100
1 y	442	36.4 (11.3)	11.9–64.9	442	46.7 (11.4)	12.9–68.9	441	59.1 (19.4)	0–100
3 y	362	36.8 (11.3)	12.2–60.9	362	47.0 (11.3)	11.4–71.9	374	57.6 (20.6)	5–100
Reference*		47.8 (10.7)			53.3 (8.7)			78.3 (17.9)	
Musculoskeletal diagnoses									
b	210	29.7 (7.2)	13.2–49.2	210	44.0 (12.1)	8.9–70.9	210	48.0 (18.3)	5–100
1 y	206	32.9 (9.6)	11.9–59.2	206	45.8 (11.7)	12.9–68.9	204	54.6 (20.2)	0–100
3 y	172	33.7 (10.0)	12.2–57.8	172	46.5 (11.6)	11.4–68.5	179	54.6 (19.8)	5–100
Circulatory diagnoses									
b	99	39.9 (9.9)	12.9–63.9	99	46.6 (11.2)	15.5–70.8	99	57.1 (17.6)	0–100
1 y	95	43.8 (11.2)	18.2–64.9	95	48.5 (10.5)	18.0–67.4	99	67.0 (17.7)	15–100
3 y	81	43.7 (11.2)	18.5–60.9	81	49.0 (9.8)	25.4–63.1	81	65.2 (18.6)	20– 96

SD: standard deviation; SF-36: Medical Outcome Study Short Form; PCS: Physical Component Summary (scale 0–100, 100 best); MCS: Mental Component Summary (0–100, 100 best); EQ-VAS: EuroQol visual analogue scale (0–100, 100 best); b: baseline; 1 y: 1-year follow-up; 3 y: 3-year follow-up.

* Norwegian reference values 50–59 years.

Participants lost to the 3-year follow-up survey were younger, female, and had a lower level of education (Table III). Further, they had a higher mean PCS score at baseline with a lower change score at 1 year, a lower mean MCS score with a larger change score, and a lower EQ-VAS score at baseline with a lower change score at 1 year compared with the participants who were not lost to follow-up (Table III).

**Table III:** Characteristics of the 72 participants lost to the 3-years follow-up survey

Category	*n*	Value
Age at baseline in years, mean (SD)	72	56.6 (14.9)
Sex (female)		40 (55.6%)
Place of residence (urban)		36 (50%)
Level of education	70	
University/college		18 (25.7%)
Upper secondary school		35 (50%)
Primary school		17 (24.3%)
Referral diagnoses	72	
Musculoskeletal		30 (41.7%)
Cardiovascular		16 (22.2%)
Neoplasm		6 (8.3%)
Neurological		3 (4.2%)
Other		17 (23.6%)
SF-36, PCS	72	
Baseline, mean (SD)		35.0 (9.6)
1-year follow-up, mean (SD)		35.9 (11.0)
SF-36, MCS		
Baseline, mean (SD)		41.4 (10.9)
1-year follow-up, mean (SD)		43.8 (10.3)
EQ-VAS	72	
Baseline, mean (SD)		48.9 (18.1)
1-year follow-up, mean (SD)		53.3 (18.9)

SD: standard deviation; SF-36: Medical Outcome Study Short Form; PCS: Physical Component Summary (scale 0–100, 100 best); MCS: Mental Component Summary (0–100, 100 best); EQ-VAS: EuroQol visual analogue scale (0–100, 100 best).

### Trajectory of PHC 3 years before and after rehabilitation

**Fig. 2:**
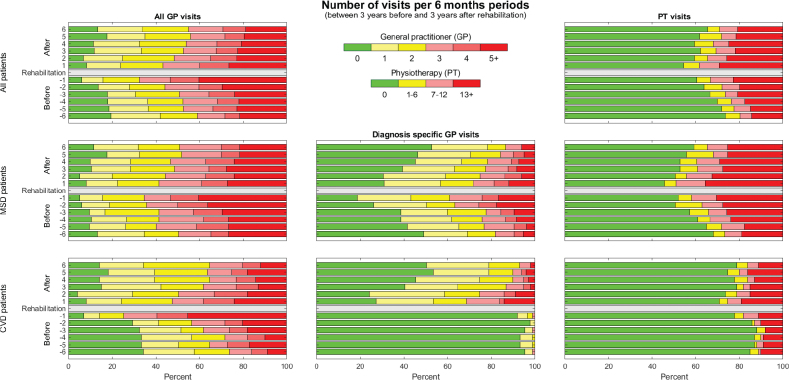
Frequency of visits to the general practitioner (GP) and physiotherapist (PT) per 6-month periods before and after rehabilitation. All GP visits (left), diagnosis specific (middle) and PT visits (right).

*GP visits.* Within the total study cohort, there was an increase in GP visits leading up to rehabilitation and a gradual decrease after rehabilitation (Fig. 2). The MSD subgroup had a similar pattern of GP visits to the total study cohort. However, the CVD subgroup had hardly any consultations specific to the diagnosis before rehabilitation, but an increase after rehabilitation when 73% had one or more visits to the GP related to the diagnosis during the first 6 months. The proportion of 1–2 visits per 6 months related to CVD remained relatively stable during the 3-year period after rehabilitation, while the proportion of patients with more frequent GP visits gradually decreased.

*PT visits.* Within the total study cohort, there was an increase in PT visits leading up to rehabilitation. After rehabilitation, there was some increase in PT visits during the first 6 months and then a gradual decrease (Fig. 2).

Stratified by diagnoses, patients with MSD had an increase in PT visits leading up to 1 year prior to rehabilitation, when 50% had one or more PT visits. After rehabilitation, a gradual decrease was found after the first 6 months. For patients with CVD, there was an increase in PT visits during the last 6 months before rehabilitation, when 22% had one or more PT visits. After rehabilitation, 30% had one or more PT visits during the first 6 months, but this later abated.

*Follow-up after rehabilitation.* We found that 54% of the total study cohort and subgroups had 1 or more GP visits during the first 30 days after rehabilitation. However, only 37% of the patients in the MSD subgroup and 40% of the patients in the CVD subgroup had visits related to the respective diagnoses within 30 days.

Furthermore, we found that 33% of the total study cohort, 43% of patients with MSD, and 18% of patients with CVD had one or more PT visits during the first 30 days after rehabilitation.

Moreover, 68% of the total study cohort, 74% of patients with MSD, and 62% of patients with CVD had either a GP or a PT visit during the first 30 days after rehabilitation. During the first 6-month period following rehabilitation, 94% of the total study cohort, and 97% and 93% of the MSD and CVD subgroups, respectively, had either a GP or a PT visit.

### Use of PHC services after rehabilitation related to self-reported health and functioning

**Fig. 3:**
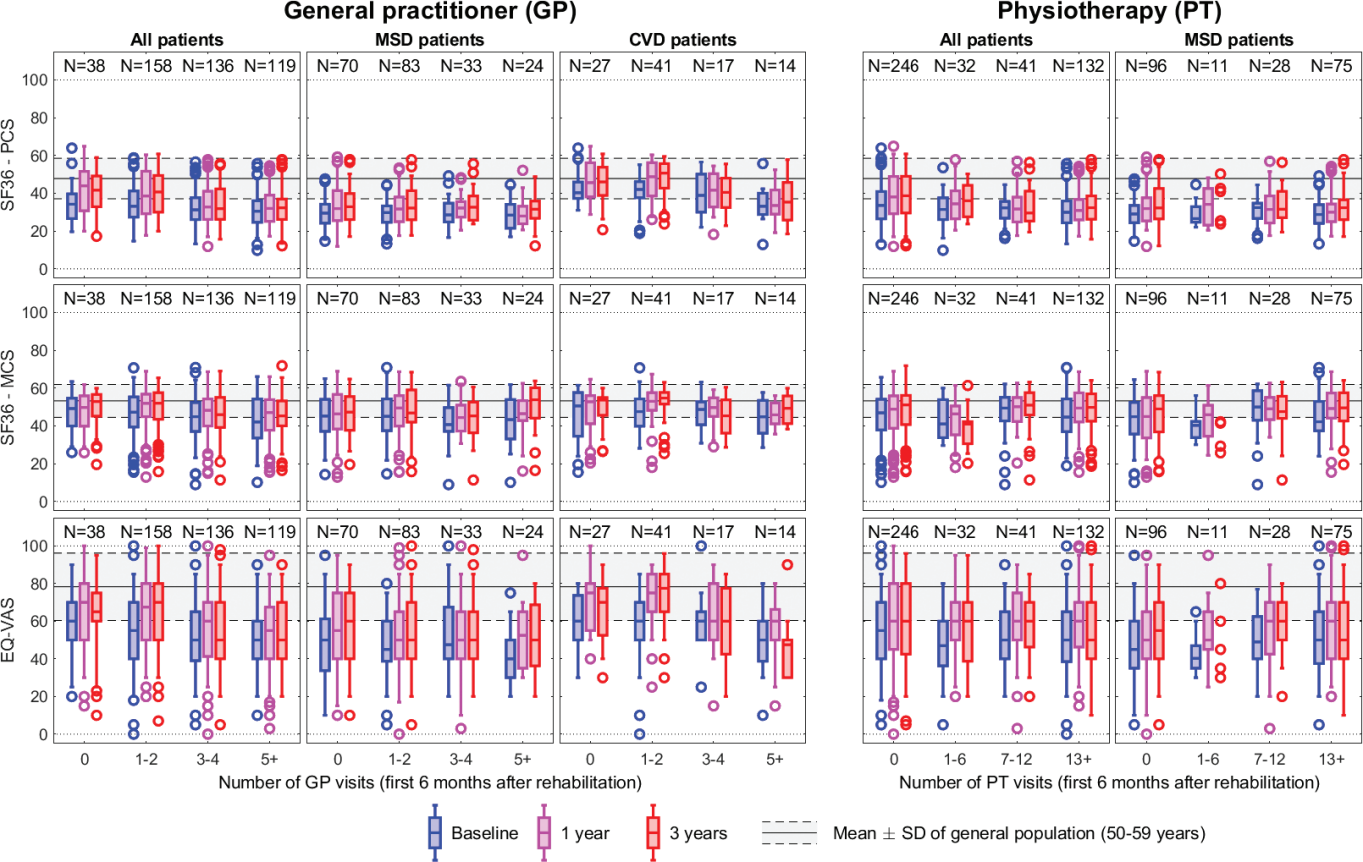
Relations between frequency of visits to the general practitioner (GP, left) and physiotherapist (PT, right) during the first 6 months following specialized rehabilitation, and physical functioning (SF36–PCS), mental functioning (SF36–MCS), and self-reported health (EQ-VAS). The Norwegian reference values are shown with 1 SD intervals. GP visits: total study cohort (left), MSD patients (middle), CVD patients (right). PT visits: total study cohort (left), MSD patients (right) (due to limited PT visits in the CVD subgroup, results are not presented).

In Fig. 3, we report the frequency of visits to the GP (left) and PT (right) during the first 6 months after rehabilitation related to measures of health (EQ-VAS) and functioning (PCS and MCS) at baseline and follow-ups at 1 and 3 years. Additionally, the Norwegian reference values (mean and SD) for the instruments are shown in the figures.

*GP visits, total study cohort.* Dispersed scores were found, particularly in the EQ-VAS. The PCS scores at baseline for the total study cohort were found to be similar across the frequency of GP visits and below the mean (SD) of the Norwegian reference data. Participants with 0–2 GP visits had a slightly higher increase in scores from baseline to follow-ups compared with participants with 3 or more visits (Fig. 3, top left). More overlap with the reference data was shown for the MCS scores at 1 and 3 years, particularly for patients with 0–2 GP visits. Participants with 3 or more visits tended to have lower scores on the EQ-VAS at both follow-ups than participants with 0–2 GP visits.

*GP visits, and MSD and CVD subgroups.* In the MSD subgroup, PCS scores at both follow-ups were similar across the frequency of GP visits (Fig. 3). Patients with more than 5 GP visits had the highest MCS scores at 3 years, being within the mean (SD) band of the Norwegian reference data. Additionally, patients with more than 5 GP visits had the lowest baseline scores in the EQ-VAS but similar scores to other frequencies of GP visits at both follow-ups.

In the CVD subgroup, patients with 1–2 GP visits had the highest scores across all measures of health and functioning and time points and similar PCS scores to patients with no GP visits (Fig. 3). Additionally, patients with 1–2 GP visits had scores of health and functioning within the mean (SD) band of the Norwegian reference data at both follow-ups.

*PT visits.* Dispersed scores were found in all measures of health and functioning and frequencies of PT visits (Fig. 3). In the total study cohort (Fig. 3) and in the MSD subgroup (Fig. 3), patients with 13 or more PT visits had the lowest PCS scores, but baseline scores were relatively similar across frequencies of PT visits.

In the CVD subgroup, patients not using PT services were found to have PCS, MCS, and EQ-VAS scores within the mean (SD) band of the Norwegian reference data at both follow-ups. However, due to the limited number of PT visits within this subgroup, the results are not presented.

### Use of PHC services after rehabilitation related to changes in self-reported health and functioning

**Fig. 4:**
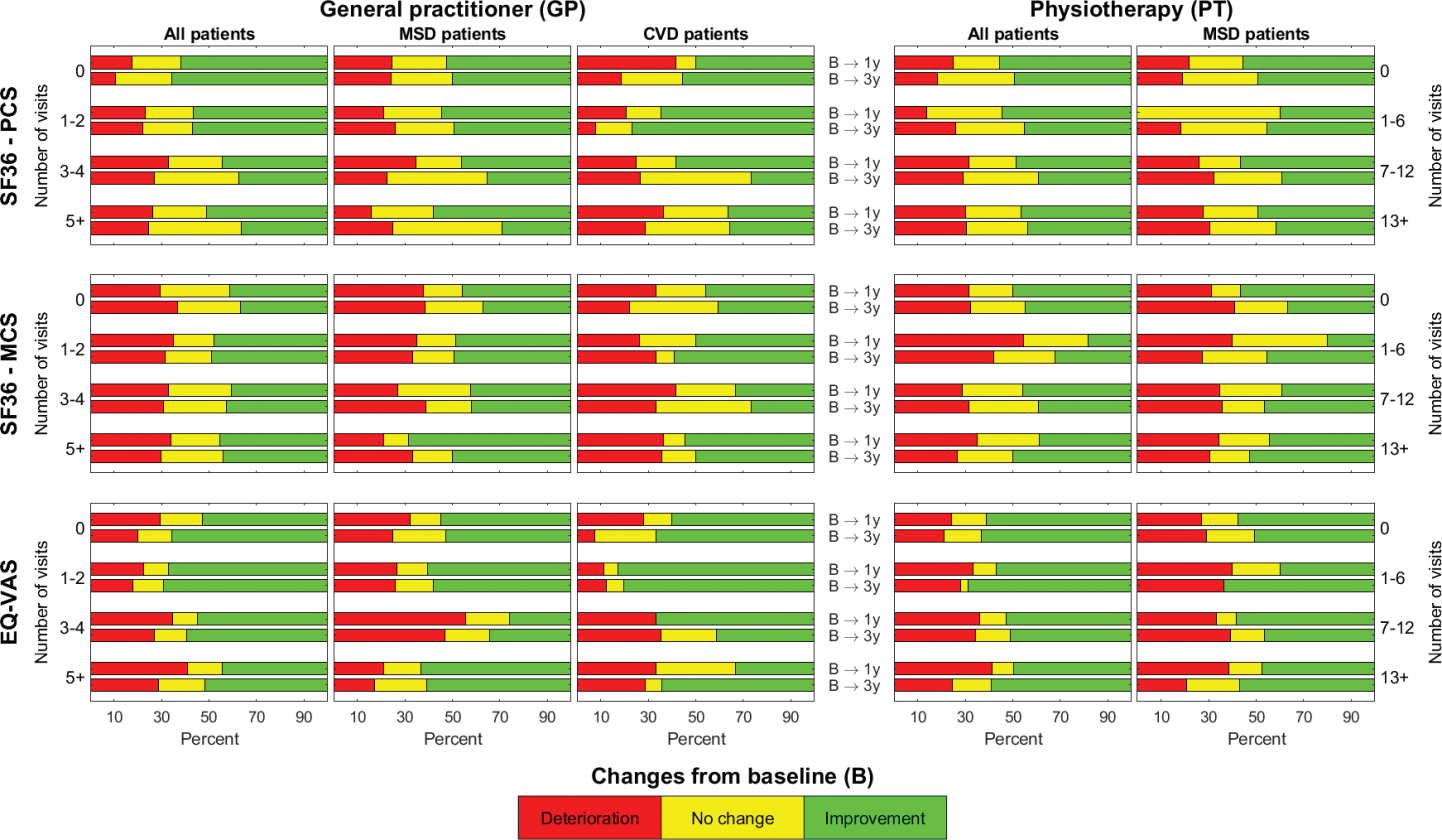
Relations between frequency of visits to the general practitioner (GP, left) and physiotherapist (PT, right) during the first 6 months following specialized rehabilitation, and change from baseline (based on minimal important values) in physical functioning (SF36–PCS), mental functioning (SF36–MCS), and self-reported health (EQ-VAS). GP visits: total study cohort (left), MSD patients (middle), CVD patients (right). PT visits: total study cohort (left), MSD patients (right) (Due to limited PT-visits in the CVD subgroup, results are not presented).

In Fig. 4, we report the frequency of GP and PT visits during the first 6 months after rehabilitation related to minimal clinically important changes in PCS, MCS and EQ-VAS from baseline to 1 and 3 years (deterioration, no change, improvement based on MIC values).

The different categories of change from baseline were found in all measures of health and functioning across all frequencies of visits to the GP (Fig. 4 right) and PT (Fig. 4 left), in both the total study cohort and subgroups.

*GP visits, total study cohort.* In the total study cohort, the change from baseline was relatively stable across time in all categories of change, especially in the MCS (Fig. 4).

*GP visits, and MSD and CVD subgroups.* In the MSD subgroup, a larger proportion of patients with 5 or more GP visits (related to the diagnosis) reported improvement in PCS (55%), MCS (65%), and EQ-VAS (60%) from baseline to 1 year rather than deterioration (15%, 20%, 20%, respectively).

In the CVD subgroup, patients with 1–2 GP visits (related to CVD) had the largest proportion of improvement in both PCS (1 year = 61%, 3 years = 75%) and EQ-VAS (1 year and 3 years = 77%) scores compared with patients with other frequencies of GP visits.

*PT visits.* In the total study cohort and the MSD subgroup, a larger proportion reported improvement compared with deterioration in PCS and EQ-VAS across all frequencies of PT visits (Fig. 4). However, in MCS, a larger proportion of patients with 1–7 PT visits reported deterioration compared with improvement.

Due to the limited number of PT visits within the CVD subgroup, the results are not presented.

## DISCUSSION

In this study, we explored the use of PHC services during the 3-year period before and after specialized rehabilitation and found an increase in GP and PT visits leading up to rehabilitation followed by a gradual decrease after rehabilitation. An exception was GP visits in the CVD subgroup, where there were few visits specific to the diagnosis before rehabilitation followed by an increase in visits after rehabilitation. It was found that 68% of the study cohort had a GP or PT visit during the first 30 days after rehabilitation. In the total study cohort, there seemed to be a trend that patients with lower levels of self-reported health and functioning had a higher frequency of PHC use after rehabilitation. A tendency for more clinically important improvement was found among those with most frequent GP visits in the MSD subgroup, and among those with 1–2 GP visits in the CVD subgroup.

### Use of services in PHC before and after rehabilitation

In our study, the need for specialized rehabilitation might be reflected in the increased use of services in PHC leading up to rehabilitation. The need was further indicated by patients’ self-reported scores of health and functioning before rehabilitation, being below the mean of the Norwegian reference group. Our study is based on a heterogeneous rehabilitation population consisting of patients with neurological diagnoses, neoplasms, in addition to MSD and CVD, being the largest subgroup. In general, considering that the MSD subgroup constituted 46% of the study cohort, we found a similar pattern of PHC use in the MSD subgroup to that in the total study cohort. However, the MSD and CVD subgroups showed different patterns; therefore, in the discussion, we chose to focus on the MSD and CVD subgroups to illustrate the diversity of the rehabilitation population and their trajectories of PHC.

We found that the MSD subgroup had a high frequency of MSD-related GP visits 3 years before (51–81%) and after (67–46%), as well as a high frequency of PT visits. Previous studies have found that GP and PT visits related to MSDs are common (8, 10, 27). In Norway, 29% of the general population had 1 or more GP visits related to MSD over a 1-year period (10). This suggests that our population, who received rehabilitation, had a higher frequency of MSD-related GP visits compared with the general population in Norway. A Danish study also found that individuals with MSD receiving rehabilitation were almost exclusively in the subgroup of frequent healthcare users (10). Considering that symptoms of (nontraumatic) MSD are often nonspecific and long-lasting, it is suggested that MSD should be managed in PHC (28). Our findings may indicate that the majority of patients with MSD are cared for within PHC as recommended. However, the increasing frequency of GP and PT visits leading up to specialized rehabilitation may reflect a need for more specialized or intensive rehabilitation than offered in PHC.

Following rehabilitation, we found that 37% of the MSD subgroup had one or more GP visits related to MSD and 43% had one or more PT visits in the first 30 days after rehabilitation. This is in line with a recent Norwegian study among patients with rheumatic and MSD based on patient-reported data (14). They found that 39% of the participants had planned follow-up with the GP and 43% with the PT following specialized rehabilitation. However, 84% reported a need for follow-up with the GP, and 83% reported a need for follow-up with the PT. Thus, the participants reported a higher need for follow-up than planned at discharge (14). Despite our finding that 97% of the MSD subgroup had one or more visits to the GP or PT during the first 6 months after rehabilitation, we argue that this does not indicate a clear trajectory of follow-up across different levels of healthcare. However, we found that visits related to MSD decreased after rehabilitation. This may indicate that the MSD conditions have improved, and the need for PHC accordingly has abated, or that they were coping better. Previous studies have found that patients with MSD who adhere to self-management activities or adapted coping strategies improved further in health and functioning after rehabilitation (29, 30). This may explain the decreasing GP visits due to MSD in our study.

In contrast, patients with CVD had hardly any GP visits related to the diagnosis prior to rehabilitation. An explanation may be that rehabilitation due to CVD is often initiated following an acute incident. We did, however, find that patients with CVD had an increasing frequency of GP visits not related to the referral diagnosis prior to rehabilitation. This finding may support a previous Dutch study that found a higher prevalence of comorbidity among patients with CVD than among patients with non-CVD diagnoses (31). Patients with CVD had an increase in GP visits related to the diagnosis after rehabilitation. The majority had 1–2 visits per 6 months during the 3-year period after rehabilitation, in line with recommendations for long-term follow-up after CVD to prevent future episodes (32). Furthermore, we found modest use of PT services after rehabilitation. However, the CVD subgroup is heterogenic, including stroke patients in addition to cardiac diagnoses. Patients followed up in PHC after cardiac rehabilitation may not need PT, in contrast to stroke survivors. High healthcare use after stroke, including benefits of long-term follow-up by the PT, is, however, well documented (15, 33).

### Use of PHC services after rehabilitation related to self-reported health and functioning

The finding that patients with more frequent use of services in PHC generally reported lower levels of health and functioning might be as expected. This study also found that some patients seemed to manage well without frequent visits after rehabilitation, while others seemed to need more to achieve higher scores of health and functioning. This may indicate that additional personal factors related to coping and lifestyle changes influence the use of PHC services. Changes in lifestyle are often found to be important for improved health and functioning for patients with both MSD (34) and CVD (33). Additionally, coping resources are found to be important for improved health and functioning in rehabilitation (12, 35). In a recent study investigating return to work, it was found that the individual’s coping resources are important for work participation after rehabilitation, especially among patients with MSD (36). Thus, the individual’s coping resources might be one of many factors influencing the frequency of PHC use after rehabilitation.

### Use of PHC services after rehabilitation related to changes in self-reported health and functioning

A varied relationship was found between change in health and functioning, and use of PHC services at follow-up. In the MSD subgroup there was a tendency for more clinically important improvement compared with deterioration among those with most frequent GP visits, whilst in the CVD subgroup, patients with 1–2 GP visits reported the greatest long-term improvement and achieved scores of health and functioning in line with the general Norwegian population. This finding may indicate the nature of the diagnoses or that some patients may need more comprehensive follow-up in PHC and still experience deterioration in health and functioning.

A Norwegian study found that GPs, along with other healthcare professionals as well as patients, associate more healthcare with better healthcare (37). However, it is suggested that excess use of healthcare threatens the health of the individual and the sustainability of healthcare systems (38). In high-income countries, there is an increasing use of healthcare that does not correlate with improved health (38). This has raised the international campaign on making wiser choices in primary healthcare (39). Thus, our finding that a higher frequency of PHC use did not appear to result in greater long-term improvement in self-reported health and functioning for all participants may add to the debate on sustainable healthcare.

Although this study offers new knowledge on PHC use before and after specialized rehabilitation related to health and functioning, we need further research to investigate how the characteristics of the individual, including coping resources, influence long-term improvement in relation to healthcare use.

### Strengths and limitations

To our knowledge, this is the first longitudinal study describing the use of PHC 3 years before and after specialized rehabilitation. A strength of this study is the use of patient-reported data in combination with register data. The longitudinal design made it possible to describe the use of PHC over a longer period than in other rehabilitation cohorts. Additionally, using validated instruments with normative data improves the external validity of the study.

This study also has several limitations, one being the relatively small sample size of the subgroups. Although the study cohort includes diagnostic groups commonly seen in somatic, specialized rehabilitation, the small sample size in each group prevents us from comparing subgroups within the study cohort, other than the MSD and CVD subgroups. It is also important to emphasize that the MSD and CVD subgroups are heterogeneous. In addition, we do not have data on specialist healthcare use after rehabilitation, and some of the patients, especially those with CVD, might have been followed-up within specialist healthcare.

### Conclusion

To summarize, we found that rehabilitation patients are frequent users of PHC services, with increasing visits to the GP and PT before rehabilitation and a gradual decrease after rehabilitation. An exception was GP visits in the CVD subgroup, with few visits related to CVD before and an increase in visits following specialized rehabilitation. Patients with lower levels of self-reported health and functioning after rehabilitation tended to have a higher frequency of PHC use. This may imply that patients who perceive low health and functioning tend to seek more frequent help and support for their complaints. The varied relationship between change in health and functioning, and use of PHC services at follow-up, may imply that additional factors besides the frequency of services used in PHC explain long-term outcome of rehabilitation.
